# High levels of follicular fluid testosterone could impair oocyte developmental competency via affecting aryl hydrocarbon receptor pathway in PCOS patients

**DOI:** 10.1186/s12860-022-00449-y

**Published:** 2022-11-11

**Authors:** Fatemeh Eini, Maryam Azizi kutenaei, Tahereh Foroutan, Ensieh Salehi

**Affiliations:** 1 Fertility and Infertility Research Center, Hormozgan University of Medical Sciences, Bandar Abbas, Iran; 2 Department of Animal Biology, Faculty of Biological Sciences, Kharazmi University, Tehran, Iran

**Keywords:** Aryl Hydrocarbon Receptor, Gene Expression, Hyperandrogenism, Polycystic Ovary Syndrome

## Abstract

**Background:**

Although hormonal and metabolic dysfunction have been recognized as a possible cause of polycystic ovarian syndrome (PCOS), the associations between hyperandrogenism and aryl hydrocarbon receptor (Ahr) signaling pathway remains controversial. The current study aimed to investigate the effect of hyperandrogenism on oocyte developmental competency via regarding Ahr signaling downstream pathway in granulosa cells.

**Materials and methods:**

Granulosa cells were collected from 45 PCOS patients under assisted reproductive technique (ART). Gene expression of Ahr downstream pathway was evaluated based on Reverse Transcription Q-PCR assay. Moreover the correlation was investigated between gene expression and hyperandrogenism, and oocyte developmental competency in PCOS.

**Results:**

From the 45 PCOS patients, 26 (64.44%) had a high level of follicular fluid testosterone (FFT). Based on the FFT level, two groups of PCOS: HFT (high level of FFT) and non-HFT, were shown significant differences in oocyte and embryo quality, and fertilization and cleavage rates. Moreover, the mean relative expressions of *Ahr* and *Arnt* genes were significantly higher in HFT –PCOS group (*p* < 0.01 and *p* < 0.01) respectively. Also, the significant positive correlations were obtained for *Ahr*, *Arnt*, *Cyp1A1, and Cyp1B1* with incidence of clinical hyperandrogenism and FFT level. Besides, our results showed that *Ahr*, *Cyp1A1*, and *Cyp1B1* gene expression was correlated significantly with fertilization rate.

**Conclusion:**

The present study suggested that hyperandrogenism could impair oocyte developmental competency via affecting Ahr signaling downstream pathway.

## Introduction

Androgens have been shown to play an essential role in normal ovarian function and folliculogenesis, as well as causing ovarian pathology [1]. In a woman's ovary, androgens via androgen receptors (AR), located in the granulosa cells, promote the growth of primary follicles and folliculogenesis [2]. In granulosa cells, androgens are converted to estrogens by the aromatase enzyme. The estrogens promote follicular growth, therefore, the development of follicles is dependent to the androgens and their receptor's balance [3, 4]. However, high levels of androgens, could also induce follicular atresia and inhibit follicle-stimulating hormone (FSH)-induced granulosa cell proliferation. Therefore, unbalanced levels of androgens could preserve analogical conditions in the ovary [5].

Polycystic ovary syndrome (PCOS) is the most common (2–27%) endocrine and metabolic disorder in reproductive-aged women characterized by hyperandrogenism and subfertility [6]. Hyperandrogenism and insulin resistance are the main factors of PCOS pathophysiology [7]. Although the PCOS pathophysiology remains to be clarified entirely, endocrine-disrupting chemicals (EDCs) by interfering in the normal endocrine function could be caused metabolic or reproductive disorders [8]. Recent studies indicated that acting EDCs, even though in a low doses, has a particular role in endocrine dysfunctions [9, 10].

Aryl hydrocarbon receptor (Ahr) is a well-established receptor for EDCs. It is a member of the growing superfamily of basic helix-loop-helix (bHLH)-PAS transcription factors, regulates the expression of a diverse established of genes, and consequently plays a significant role in various metabolic, developmental, and pathologic processes [11, 12]. Activation of Ahr signaling pathway controls the physiological functions of the reproductive organs including ovaries, oviducts, uterus, and vagina [13]. However, the Ahr downstream signaling is regulated by several stimuli and inhibitory factors like growth factors and hormones even though without its ligands [14, 15].

The correlation between androgens with Ahr cascade has not been thoroughly examined. It has been indicated that androgens via affecting the Ahr cascade could promote or inhibit the apoptotic process in granulosa cells [16]. This process is completely dependent to the kind of the androgen. It was reported that some of androgens such as testosterone prompt the expression of Ahr and its interaction with the androgen receptor. Also they showed that testosterone could stimulate Ahr expression and the interaction between Ahr and androgen receptor (Ar), leading to the stimulation of the liver receptor homolog 1 (LRH-1) expression in rat granulosa cells. Outlandishly, this effect was not represented by dihydrotestosterone (DHT) [16].

To date however, a clear association of androgen with Ahr and downstream signaling expression in PCOS women has not been proven. Therefore, we hypothesized that expression of Ahr and stimulation of its downstream signaling pathway could be impacted by androgens in granulosa cells and contribute to PCOS pathology, regardless of the presence of EDCs. To this, we analyzed the expression of *Ahr* and Ahr nuclear translocator (*Arnt*) and *Cyp1A1* and *Cyp1B1* in granulosa cells of PCOS patients. Configuration of Ahr/Arnt heterodimer is essential for activation of downstream signaling pathway, including Cytochrome P450 family. Both *Cyp1A1* and *Cyp1B1* belong to superfamily of Cytochrome P450 (CYP1) enzymes which have a critical role in the synthesis and metabolism of steroid hormones in the ovary. Based on all these data the aim of our study was to investigate the correlation between the follicular testosterone and Ahr cascade expression genes and ovarian stimulation outcomes in female population with PCOS.

## Material and methods

### Patients

A total of 45 PCOS patients referred in the fertility and infertility center of Hormozgan University of medical sciences were participated in this study. All the participants signed the written informed consent and the study was approved by the mentioned University with the ethical committee code: IR.HUMS.REC.1398.436.

The Rotterdam criteria was used for detection of PCOS patient. The patients with the at least 2 of the following criteria were included in this study: Oligomenorrhea which was defined as less than 8 periods per year or cycles longer than 35 days, and secondary amenorrhea was defined as no menstruation for more than 90 days. Clinical hyperandrogenism was defined by a modified Ferriman and Gallwey score (mF-G score) of six or greater [17] and the polycystic ovary was defined as the presence of 12 or more follicles measuring 2–9 mm in diameter, or an increased ovarian volume (exceeding 10 ml) [18]. However, the woman with other disorders that mimic the features of PCOS, ovarian tumors, adrenal disorders were excluded from the study. Moreover, women who had taken oral contraceptives, lipid-lowering agents, and insulin sensitizers in the 3 months preceding the start of the study were also excluded. All the woman had normal uterus structures and their partners had normal spermiograms.

Based on the high level of follicular testosterone > 3 (HFT) the 45 PCOS patients divided into HFT-PCOS group (*n* = 29) and non-HFT PCOS group (*n* = 16). All the demographic characteristic (age, body mass index (BMI), basal FSH and LH) were similar in the each groups. The characteristic data was showed in the Table 1.

**Table 1:** The clinical and hormonal characteristics of PCOS patients

Groups	Non-HFT-PCOS	HFT-PCOS	*P* value
Age (years)	30.14 ± 2.28	29.43 ± 2.73	NS: 0.84
BMI (kg/m2)	26.57 ± 0.64	25.29 ± 0.61	NS: 0.93
FSH (mIU/ml)	6.28 ± 0.56	6.42 ± 0.28	NS: 0.78
LH (mIU/ml)	7.93 ± 0.55	7.14 ± 0. 46	NS: 0.39
AMH	4.04 ± 0.28	4.71 ± 0.43	NS: 0.15
PRL	15.01 ± 1.12	14.94 ± 1.09	NS: 0.78
Days of ovarian stimulation	10.12 ± 1.67	10.54 ± 1.13	NS: 0.64
Total gonadotropin dose (IU)	2054 ± 345	1970 ± 278	NS: 0.48
Modified F-G Score	7.45 ± 1.17	8.01 ± 1.09	NS: 0.22
FFT (ng/ml)	3.41 ± 0.38	5.14 ± 0.42	0.01*
AFC	18.86 ± 1.22	17.42 ± 1.39	NS:0.46
GV oocytes (No)	2.86 ± 0.41	4.85 ± 0.67	0.025*
MI oocytes	1.53 ± 0.26	1.64 ± 0.44	NS:0.99
MII oocytes	11.86 ± 0.86	9.71 ± 0.97	0.044*
Fertilization rate (%)	76.14 ± 3.81	64.57 ± 3.57	0.039*
Cleavage rate (%)	72.43 ± 3.22	62.15 ± 3.61	0.032*
Grade A embryo (No)	7 .00 ± 049	5.43 ± 0.52	0.049*
Grade C embryo (No)	4.71 ± 0.42	5.00 ± 0.62	NS: 0.71

The data were presented as mean ± SEM

*HFT* high follicular fluid testosterone, *PCOS* polycystic ovary syndrome, *BMI* body mass index, *FSH* follicle-stimulating hormone, *LH* luteinizing hormone, *PRL* prolactin, *AMH* anti-Mullerian hormone, *F-G Score* Ferriman and Gallwey score, *FFT* follicular fluid testosterone, *AFC* antral follicular count, *GV* germinal vesicle, *MI* metaphase I, *MII* metaphase II

^*^Significance values

### Ovarian stimulation

Granulosa cells were obtained from the PCOS patients undergoing assistant reproductive technique. A GnRH antagonist protocol was used for ovarian stimulation and oocyte retrieval. Briefy, 150 IU rFSH (Gonal-f; Merck Serono) was administered from the third day of the cycle. When follicles of > 12 mm were observed, the GnRH antagonist (0.25 mg Cetrotide, Merck Serono) was initiated and continued up to human chorionic gonadotropin (hCG) injection. The follicular growth was daily monitored using ultrasound. A dose of 10,000 IU hCG (Ovitrelle, Merck Serono) was injected when at least three follicles of ≥ 18 mm were observed. Oocyte retrieval was carried out transvaginally under ultrasound guidance 36–40 h after hCG administration [19].

### Isolation and collection of cumulus cells and follicular fluid

After cumulus-oocyte complexes (COCs) picking up, the follicular fluid was collected for hormonal assay and cumulus cells were isolated by gently stripping using hyaluronidase (Life Global, India). The cumulus cells of mature oocytes were pooled and transferred into micro-centrifuge tubes. Then, they were washed with PBS using centrifuge. After twice washing 150 µl RNA stabilization reagent buffer (RLT, Qiagen, the Netherlands) was added to pellet of cells. Finally, the cells were stored at − 80 °C for RNA extraction.

### RNA isolation and quantitative real-time polymerase chain reaction

The total RNA was manually isolated from the cumulus cells using Trizol reagent (Sigma Pool, UK) following the manufacturer’s protocol. The transcript nova kit (Qiagen Inc., Valencia, CA, USA) was used for reverse-transcription of obtained RNA into cDNA as a described previously [20]. The reverse-transcribed yields of *Ahr*, *Arnt*, *Cyp1A1*, and *Cyp1B1*, were amplified by real-time polymerase chain reaction (PCR) with SYBR Green (Takara, Japan) on an ABI real-time PCR system (Applied Biosystems, ABI, Foster City, CA, USA), according to the manufacturer’s instructions. Finally, all data were analyzed by the standard formula, while glyceraldehyde-3-phosphate dehydrogenase (*Gapdh*) was used as an internal reference gene. Primer sequences were as follows: human AHR (sense, 5´-AGAGTTGGACCGTTTGGCTA-3´; antisense, 5´AGTTATCCTGGCCTCCGTTT-3´), human *ARNT* (sense, 5´- CAAGCCCCTTGAGAAGTCAG-3´; antisense, 5´-GGGGTAGGAGG GAATGTGTT-3´), human *CYP1A1* (sense, 5′-TCA ATC AAG AGG CGC GAA CCT C-3′; antisense, 5′-CTA CAG CCT ACC AGG ACT CG-3′, human *CYP1B1* (sense, 5´-AAGTTCTTGAGGC ACTGCGAA-3´; antisense, 5´-GGCCGGTACGTTCTCCAAAT-3´), and human GAPDH (sense, 5´-TGGACCTGACCTGCCGTCTA-3´; antisense, 5´-CTGCTTCACCACCTTCTTGA-30).

### Quality assessment of zygote and embryo development

Fertilization was assessed by observation of two pronuclei 16–18 h after sperm injection according to Scott et al. criteria [21]. Moreover, the embryonic development competence was evaluated based on the amount of the fragmentation and the count of blastomeres on day 3. According to Depa-Martynow et al. four scores were given to the developed embryos [22]. Grade A (high quality): embryo with 7–9 blastomeres and cytoplasmic fragmentation up to 20%; Grade B: embryos with 7–9 blastomeres and the cytoplasmic fragmentation more than 20%; Grade C: 4–6 cell embryos with a cytoplasmic fragmentation of up to 20%; Grade D (low quality): 4–6 cell embryos and the cytoplasmic fragmentation of more than 20%.

### Statistical analysis

The statistical analysis of the results was carried out using Graphpad Prism 6.04 ((Graphpad Software, Inc., San Diego, CA). Student’s *t*-test was used to analyze all measurements between two groups of PCOS. The correlations were measured using Pearson’s correlation coefficients. The differences among the groups were considered statistically significant when the *p*-value was < 0.05.

## Results

### Basic characteristics of the participants and the clinical outcomes

All the demographic and clinical characteristics of the PCOS patients with or without HFT are listed in Table 1. No significant differences were observed in the age, BMI, FSH, LH, AMH, PRL, days of ovarian stimulation, gonadotropin dose, AFC count, and modified f-G Score from two groups. However, the significant differences was showed in follicular fluid testosterone, oocyte and embryo quality, and fertilization and cleavage rates between two groups.

### mRNA expressions of Ahr, Arnt, and Cyp1A1 and Cyp1B1 in the granulosa cells

**Fig. 1:**
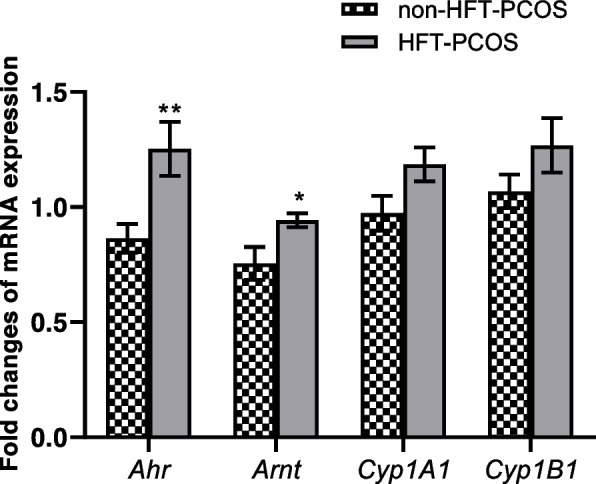
The Relative mRNA expressions of Ahr signaling downstream pathway genes in PCOS patients. Data are the mean ± SEM. *P < 0.05, and**P < 0.01

The results showed that the mean relative expressions of *Ahr* and *Arnt* genes were significantly higher in PCOS patients with the high levels of follicular testosterone levels. Although, the mean expression of both *Cyp1A1* and *Cyp1B1* were higher in in PCOS patients with the high levels of follicular testosterone levels, however these results were not significantly different (Fig. 1).

### Correlation of gene expressions with Androgen levels and oocyte developmental competency in the granulosa cells of PCOS patients

The results of our study showed that in PCOS patients, the significant positive correlations were obtained for *Ahr*, *Arnt*, *Cyp1A1, and Cyp1B1* with incidence of clinical hyperandrogenism (Table 2). Moreover, significant correlation were found between the levels of *Ahr* and *Cyp1B1* mRNA expression and follicular fluid testosterone level (Table 2). Also, we observed correlation between *Ahr*, *Cyp1A1*, and *Cyp1B1* gene expression and fertilization rate (Table 2).

**Table 2:** Pearson correlation coefficients between gene expression and clinical and hormonal characteristics in PCOS patients

	**Gene expression**			
Characteristics	***Ahr***	***Arnt***	***Cyp1A1***	***Cyp1B1***
Modified F-G Score	*r* = 0.305 *p* = 0.04*	*r* = 0.451 *p* = 0.015*	*r* = 0.341 *p* = 0.28	*r* = 0.276 *p* = 0.044*
FFT	*r* = 0.372 *p* = 0.020*	*r* = 0.159 *p* = 0.157	*r* = 0.276 *p* = 0.053	*r* = 0.596 *p* = 0.001*
Fertilization rate (%)	*r* = 0.718 *p* = 0.001*	*r* = 0.130 *p* = 0.203	*r* = 0.315 *p* = 0.041	*r* = 0.706 *p* = 0.006*
Cleavage rate (%)	*r* = 0.314 *p* = 0.544	*r* = 0.56 *p* = 0.411	*r* = 0.014 *p* = 0.681	*r* = 0.106 *p* = 0.255
Grade A embryo	*r* = 0.381 *p* = 0.018	*r* = 0.114 *p* = 0236	*r* = 0.188 *p* = 0.12	*r* = 0.196 *p* = 0.118
Grade B embryo	*r* = 0.121 *p* = 0.221	*r* = 0.316 *p* = 0.236	*r* = 0.037 *p* = 0.506	*r* = 0.066 *p* = 0.412

*F-G Score* Ferriman and Gallwey score, *FFT* follicular fluid testosterone

^*^Significance values

## Discussion

There are several mechanisms that could affect folliculogenesis in PCOS patients, however, the relationship between hyperandrogenism and Ahr signaling pathway remains unclear. In the present study, the Ahr signaling pathway was investigated in PCOS patients to explore the effect of androgens on oocyte developmental competency with a consideration of Ahr signaling. Our results showed a high level of follicular fluid testosterone in PCOS patients with or without clinical hyperandrogenism. However, lower oocyte and embryo quality and fertilization rate were showed in HFT-PCOS group in comparison with non-HFT-PCOS. As the results showed that overexpression of *Ahr* and *Arnt* mRNA, in turn, increased the mRNA expression of *Cyp1B1* in the granulosa cells of HFT-PCOS group. Also, the results showed a significant correlation between clinical hyperandrogenism and FF testosterone and Ahr signaling pathway. Similarly, the correlation between increases in *Ahr* expression and oocytes developmental competency revealed that androgens via affecting Ahr signaling could deteriorate oocyte and embryos quality in PCOS patients.

Here we showed that 64.44% of PCOS patients with or without clinical hyperandrogenism displayed excessive level of FF testosterone. These results are consistent with the Li et al. study which indicated that some PCOS patients showed high level of FF androgen despite normal circulating androgen levels. Their finding showed that increased in ovarian androgen are independent of circulating androgen, therefore, indicated that ovarian hyperandrogenism could be more important for ovarian malfunctions [23]. Hence, in this study based on the high level of ovarian testosterone level, PCOS patients were divided into two groups: HFT-PCOS and non HFT-PCOS groups.

Our findings showed that low oocyte and embryo quality in HFT-PCOS group in comparison with non-ones. Hyperandrogenism has been confirmed negatively affected oocyte and embryo quality via different mechanisms in PCOS patients [24]. For instance, excessive androgen level could stimulate production of reactive oxygen species (ROS) in PCOS patients [25]. Accumulation of intracytoplasmic ROS level in oocyte and granulosa cells are associated with disturbance of glutathione production and subsequently oocyte cytoplasmic maturation [25]. Also, the recent study by Kunitomi et al. indicated that hyperandrogenism via endoplasmic reticulum (ER) stress by activating of Ahr signaling pathway contributes to PCOS pathology [8]. However, the effect of Ahr signaling pathway on oocyte maturation and granulosa cells is steel unclear and needs to be more investigated.

The cross talk between oocyte and its surrounding granulosa cells has an essential role in oocyte maturation and development [26]. Therefore, any disturbance or abnormality in gene expression and signaling pathway of granulosa cells could affect oocyte developmental competency [27, 28]. Molecular analysis suggests that androgens could impact gene expression in PCOS oocytes and granulosa cells [16]. Interestingly, Ahr and Arnt are identified to interact with androgen receptor [9]. Therefore, it seems that hyperandrogenism could affect Ahr signaling pathway. Our study showed that *Ahr* and its downstream signaling were overexpressed in the granulosa cells of HFT-PCOS group. Moreover, a positive correlation was observed between follicular fluid testosterone and Ahr mRNA expression and subsequently its downstream pathway, *Cyp1B1,* in granulosa cells, may cause deteriorative effect in oocyte quality rate and fertilization rate.

Originally, binding of Ahr to EDCs including, polychlorinated dibenzodioxins (dioxins), polychlorinated biphenyls (PCBs), and polycyclic aromatase hydrocarbons (PAHs), activates its downstream pathway [29]. Studies showed that activation of Ahr downstream pathway overexpresses Arnt, CypA1/A2, and Cyp1B1 in the human ovaries [8, 13]. The binding of Ahr as a receptor to its ligand causes the translocation of Ahr from the cytoplasm to the nucleus for configuration of Ahr/Arnt heterodimer. This heterodimer binds to xenobiotic responsive elements (XREs) to stimulate target gens [15]. CYP1A1/A2 and CYP1B1 have been shown as targets of Ahr activation. These genes belong to the superfamily of Cytochrome P450 (CYP1) enzymes which have a critical role in the synthesis and metabolism of steroid hormones and their related pathways. Also, the CYP1 enzymes are associated with the pathogenesis of several diseases and syndrome along with some drugs function [30].

Studies revealed that Ahr and downstream signaling has a crucial role in PCOS pathogenesis [8, 18]. It has been shown that the Ahr ligands are increased in the serum level of PCOS when compared with healthy women. These increases are correlated with the hormonal and metabolic disturbance in PCOS patients [8]. In addition, some findings identified several endogenous Ahr ligands which along with the independent role of Ahr as an EDC receptor have an essential role in PCOS pathogenesis. It seems that Ahr pathway could be independently activated without endogenous or exogenous ligands in PCOS patients [31, 32].

Our findings showed that *Cyp1A1*, and *Cyp1B1* mRNA expression were upregulated in the granulosa cells of PCOS patients. Moreover, we showed that there is a correlation between clinical hyperandrogenism and follicular testosterone levels and *Cyp1A1,* and *Cyp1B1* mRNA levels. Recent studies revealed that ovarian steroid hormones could impact the activation of the Ahr pathway even in the lack of its ligands [8, 33].

In the theca cells of the ovaries, 17β-hydroxysteroid dehydrogenase can convert androstenedione to testosterone which diffuses into the granulosa cells. Then, following follicle-stimulating hormone (FSH) stimulus, testosterone can be converted to 17β-estradiol by the aromatase enzyme [34]. Estradiol has an essential role in oocyte maturation and follicular development. However, estradiol can be metabolized into 2-hydroxyestradiol by Cyp1A1/A2, or to 4-hydroxyestradiol by Cyp1B1. These alterations can cause the inactivation of estradiol [35, 36] (Hayes et al. 1996; Tsuchiya et al. 2005). Therefore, it seems that overexpression of *CYP1A1*, *CYP1A2*, and *CYP1B1* could inhibit estradiol activation and its effect on folliculogenesis. In this regard, there are a few studies about Ahr mechanisms in the granulosa cells of PCOS patients. The findings of Bussmann and Barañao showed that Ahr expression was decreased in the granulosa cells when they are cultured with FSH or estradiol hormones [37]. Another study indicated that both FSH and LH are required for activation of Ahr signaling in murine granulosa cells. Moreover, they showed that protein kinase A (PKA) signaling could down-regulate the Ahr expression [29].

Based on the finding of this study, it seems that high follicular fluid testosterone levels could stimulate the expression of Ahr at the mRNA levels and signaling pathway. Consistent with our finding Wu et al. indicated that testosterone stimulates the expression of Ahr and the formation of Ar/Ahr complex [16]. Moreover similar to these findings Kunitomi et al. revealed that in the granulosa cells ER stress up-regulates Ahr expression. In this regard, some studies indicated that the activation of ER stress in the granulosa cells could have one of the main causes of PCOS pathology. Noticeably, they showed that testosterone activates ER stress in the granulosa cells. Collaboration of hyperandrogenism and ER stress stimulate Ahr signaling in granulosa cells which has an essential role in PCOS pathology [8].

Our results showed the low quality of oocytes and embryos and subsequently low fertilization rate in HFT-PCOS patients when compared with the non-HFT-PCOS group. Moreover, we showed a correlation between fertilization rate and *Ahr*, *Cyp1A1*, and *Cyp1b1* mRNA levels. This suggests that the high level of follicular androgens significantly increases transcription levels of *Ahr*, *Cyp1A1*, and *Cyp1b1* genes and disturb folliculogenesis. Therefore, low oocyte and embryo quality and subsequent fertilization rate should not be unexpected in this group. In this regard, Kunitomi et al. suggested that administration of CH223191 inhibits *Ahr* expression and could be considered as a novel therapeutic method for PCOS patients [8]. Studies showed that high level of *Cyp1B1* mRNA expression in granulosa cells are associated with PCOS pathology [38]. Cyp1B1 converts estradiol to its inactive metabolites. Therefore low level of estradiol could affect folliculogenesis in PCOS patients [39].

In conclusion, our results are indicating that the high level of follicular fluid testosterone could impair oocyte developmental competency via affecting transcription levels of Ahr signaling downstream genes in PCOS patients.

## Data Availability

The datasets used and/or analyzed during the current study are available from the corresponding author upon reasonable request.

## References

[1] Baptiste CG, Battista M-C, Trottier A, Baillargeon J-P (2010). Insulin and hyperandrogenism in women with polycystic ovary syndrome. J Steroid Biochem Mol Biol.

[2] Xing C, Zhang J, Zhao H, He B (2022). Effect of sex hormone-binding globulin on polycystic ovary syndrome: mechanisms, manifestations, genetics, and treatment. Int J Women's Health.

[3] Emami N, Moini A, Yaghmaei P, Akbarinejad V, Shahhoseini M, Alizadeh AR (2021). Differences in expression of genes related to steroidgenesis in abdominal subcutaneous adipose tissue of pregnant women with and without PCOS; a case control study. BMC Pregnancy Childbirth.

[4] Kumari S, Chaurasiya V, Onteru SK, Singh D (2021). Regulation of granulosa cell functions through NRP-1 mediated internalization of follicular fluid non-exosomal miR-210. Cell Tissue Res.

[5] Colella M, Cuomo D, Peluso T, Falanga I, Mallardo M, De Felice M, Ambrosino C (2021). Ovarian aging: role of pituitary-ovarian axis hormones and ncRNAs in regulating ovarian mitochondrial activity. Front Endocrinol.

[6] Escobar-Morreale HF (2018). Polycystic ovary syndrome: Definition, aetiology, diagnosis and treatment. Nat Rev Endocrinol.

[7] AziziKutenaei M, Hosseini Teshnizi S, Ghaemmaghami P, Eini F, Roozbeh N (2021). The effects of myo-inositol vs metformin on the ovarian function in the polycystic ovary syndrome: a systematic review and meta-analysis. Eur Rev Med Pharmacol Sci.

[8] Kunitomi C, Harada M, Kusamoto A, Azhary JM, Nose E, Koike H, Xu Z, Urata Y, Takahashi N, Wada-Hiraike O (2021). Induction of aryl hydrocarbon receptor in granulosa cells by endoplasmic reticulum stress contributes to pathology of polycystic ovary syndrome. Mol Hum Reprod.

[9] Montazeri-Najafabady N, Chatrabnous N, Arabnezhad MR, Azarpira N (2021). Anti-androgenic effect of astaxanthin in LNCaP cells is mediated through the aryl hydrocarbon-androgen receptors cross talk. J Food Biochem.

[10] Bajard L, Negi CK, Mustieles V, Melymuk L, Jomini S, Barthelemy-Berneron J, Fernandez MF, Blaha L (2021). Endocrine disrupting potential of replacement flame retardants – Review of current knowledge for nuclear receptors associated with reproductive outcomes. Environ Int.

[11] Pocar P, Fischer B, Klonisch T, Hombach-Klonisch S (2005). Molecular interactions of the aryl hydrocarbon receptor and its biological and toxicological relevance for reproduction. Reproduction.

[12] Swedenborg E, Rüegg J, Mäkelä S, Pongratz I (2009). Endocrine disruptive chemicals: Mechanisms of action and involvement in metabolic disorders. J Mol Endocrinol.

[13] Baba T, Mimura J, Nakamura N, Harada N, Yamamoto M, Morohashi K-I, Fujii-Kuriyama Y (2005). Intrinsic function of the aryl hydrocarbon (dioxin) receptor as a key factor in female reproduction. Mol Cell Biol.

[14] Harper PA, Riddick DS, Okey AB (2006). Regulating the regulator: Factors that control levels and activity of the aryl hydrocarbon receptor. Biochem Pharmacol.

[15] Larigot L, Juricek L, Dairou J, Coumoul X (2018). AhR signaling pathways and regulatory functions. Biochimie Open.

[16] Wu Y, Baumgarten SC, Zhou P, Stocco C (2013). Testosterone-dependent interaction between androgen receptor and aryl hydrocarbon receptor induces liver receptor homolog 1 expression in rat granulosa cells. Mol Cell Biol.

[17] Kim JJ, Chae SJ, Choi YM, Hwang SS, Hwang KR, Kim SM, Yoon SH, Moon SY (2011). Assessment of hirsutism among Korean women: Results of a randomly selected sample of women seeking pre-employment physical check-up. Hum Reprod.

[18] Chung YK, Kim JJ, Hong MA, Hwang KR, Chae SJ, Yoon SH, Choi YM (2021). Association between polycystic ovary syndrome and the polymorphisms of aryl hydrocarbon receptor repressor, glutathione-S-transferase T1, and glutathione-S-transferase M1 genes. Gynecol Endocrinol.

[19] Mohammadi S, Eini F, Bazarganipour F, Taghavi SA, Kutenaee MA (2021). The effect of Myo-inositol on fertility rates in poor ovarian responder in women undergoing assisted reproductive technique: a randomized clinical trial. Reprod Biol Endocrinol.

[20] Diaz F, O’brien M, Wigglesworth K, Eppig J (2006). The preantral granulosa cell to cumulus cell transition in the mouse ovary: development of competence to undergo expansion. Dev Biol.

[21] Scott L, Alvero R, Leondires M, Miller B (2000). The morphology of human pronuclear embryos is positively related to blastocyst development and implantation. Hum Reprod.

[22] Depa-Martynow M, Jedrzejczak P, Pawelczyk L (2007). Pronuclear scoring as a predictor of embryo quality in in vitro fertilization program. Folia histochemica et cytobiologica.

[23] Li A, Zhang L, Jiang J, Yang N, Liu Y, Cai L, Cui Y, Diao F, Han X, Liu J (2018). Follicular hyperandrogenism and insulin resistance in polycystic ovary syndrome patients with normal circulating testosterone levels. J Biomed Res.

[24] Eini F, Novin MG, Joharchi K, Hosseini A, Nazarian H, Piryaei A, Bidadkosh A (2017). Intracytoplasmic oxidative stress reverses epigenetic modifications in polycystic ovary syndrome. Reprod Fertil Dev.

[25] Eini F, Bidadkosh A, Nazarian H, Piryaei A, Ghaffari Novin M, Joharchi K (2019). Thymoquinone reduces intracytoplasmic oxidative stress and improves epigenetic modification in polycystic ovary syndrome mice oocytes, during in-vitro maturation. Mol Reprod Dev.

[26] Jafarzadeh H, Nazarian H, Ghaffari Novin M, Shams Mofarahe Z, Eini F, Piryaei A (2018). Improvement of oocyte in vitro maturation from mice with polycystic ovary syndrome by human mesenchymal stromal cell–conditioned media. J Cell Biochem.

[27] Shirzeyli MH, Amidi F, Shamsara M, Nazarian H, Eini F, Shirzeyli FH, Zolbin MM, Novin MG, Joupari MD (2020). Exposing mouse oocytes to mitoq during in vitro maturation improves maturation and developmental competence. Iran J Biotechnol.

[28] Li J, Chen H, Gou M, Tian C, Wang H, Song X, Keefe DL, Bai X, Liu L (2021). Molecular features of polycystic ovary syndrome revealed by transcriptome analysis of oocytes and cumulus cells. Front Cell Dev Biol.

[29] Matvere A, Teino I, Varik I, Kuuse S, Tiido T, Kristjuhan A, Maimets T (2019). Fsh/lh-dependent upregulation of ahr in murine granulosa cells is controlled by pka signaling and involves epigenetic regulation. Int J Mol Sci.

[30] Chapkin RS, Davidson LA, Park H, Jin UH, Fan YY, Cheng Y, Hensel ME, Landrock KK, Allred C, Menon R (2021). Role of the Aryl Hydrocarbon Receptor (AhR) in mediating the effects of coffee in the colon. Mol Nutr Food Res.

[31] Murray IA, Patterson AD, Perdew GH (2014). Aryl hydrocarbon receptor ligands in cancer: Friend and foe. Nat Rev Cancer.

[32] Bock KW (2020). Aryl hydrocarbon receptor (AHR) functions: Balancing opposing processes including inflammatory reactions. Biochem Pharmacol.

[33] Shivanna B, Chu C, Moorthy B (2022). The Aryl Hydrocarbon Receptor (AHR): a novel therapeutic target for pulmonary diseases?. Int J Mol Sci.

[34] Rosenfield RL, Ehrmann DA (2016). The Pathogenesis of Polycystic Ovary Syndrome (PCOS): the hypothesis of PCOS as functional ovarian hyperandrogenism revisited. Endocr Rev.

[35] Hayes CL, Spink DC, Spink BC, Cao JQ, Walker NJ, Sutter TR (1996). 17 beta-estradiol hydroxylation catalyzed by human cytochrome P450 1B1. Proc Natl Acad Sci U S A.

[36] Tsuchiya Y, Nakajima M, Yokoi T (2005). Cytochrome P450-mediated metabolism of estrogens and its regulation in human. Cancer Lett.

[37] Bussmann UA, Barañao JL (2006). Regulation of aryl hydrocarbon receptor expression in rat granulosa cells. Biol Reprod.

[38] Haouzi D, Assou S, Monzo C, Vincens C, Dechaud H, Hamamah S (2012). Altered gene expression profile in cumulus cells of mature MII oocytes from patients with polycystic ovary syndrome. Hum Reprod.

[39] Jarrett BY, Brink HV, Oldfield AL, Lujan ME (2020). Ultrasound Characterization of Disordered Antral Follicle Development in Women with Polycystic Ovary Syndrome. J Clin Endocrinol Metab.

